# Perioperative Management of Biologic and Targeted Synthetic DMARDs in Orthopedic Surgery: Balancing Infection Risk and Disease Control

**DOI:** 10.3390/microorganisms14020398

**Published:** 2026-02-07

**Authors:** Francesco Mancuso, Jacopo Angelini, Alen Zabotti, Francesco Russiani, Massimo Baraldo, Luca Quartuccio, Hemant Pandit, Paolo Di Benedetto, Araldo Causero

**Affiliations:** 1 Orthopaedic Clinic, University Hospital Friuli Centrale Azienda Sanitaria Universitaria Friuli Centrale “ASUFC”, 33100 Udine, Italy; francesco.mancuso@uniud.it (F.M.); paolo.dibenedetto@uniud.it (P.D.B.); araldo.causero@uniud.it (A.C.); 2 Department of Medicine (DMED), University of Udine (UNIUD), 33100 Udine, Italy; massimo.baraldo@uniud.it (M.B.); luca.quartuccio@uniud.it (L.Q.); 3 Clinical Pharmacology and Toxicology Institute, University Hospital Friuli Centrale Azienda Sanitaria Universitaria Friuli Centrale “ASUFC”, 33100 Udine, Italy; francesco.russiani@asufc.sanita.fvg.it; 4 Rheumatology Division, University Hospital Friuli Centrale Azienda Sanitaria Universitaria Friuli Centrale “ASUFC”, 33100 Udine, Italy; alen.zabotti@asufc.sanita.fvg.it; 5 Department of Medicine, Surgery and Health Sciences, University of Trieste, 34149 Trieste, Italy; 6 Leeds Institute of Rheumatic and Musculoskeletal Medicine, University of Leeds, Chapel Allerton Hospital, Chapeltown Road, Leeds LS7 4SA, UK; h.pandit@leeds.ac.uk

**Keywords:** DMARDs, orthopedic infections, immunomodulatory therapies, chronic inflammatory arthritis, Clinical Pharmacology

## Abstract

The perioperative management of biologic and immunomodulatory therapies in patients undergoing orthopedic surgery poses a clinical challenge, primarily due to the increased risk of postoperative infections. Biologic agents, particularly TNF inhibitors and interleukin-targeting drugs, may impair host immune responses, potentially increasing the risk of surgical site infections (SSIs), delayed wound healing, and systemic infections. However, abrupt discontinuation of these therapies can lead to disease flare-ups, which themselves may complicate recovery and rehabilitation. In addition, discontinuation of biologics can lead to drug tolerance and unresponsiveness when they are restarted and thereby need switching to another biologic. Recent studies suggest that the infection risk is particularly elevated with ongoing biologic therapy during major surgeries, especially in procedures involving prosthetic implants. Guidelines generally recommend withholding biological disease-modifying antirheumatic drugs (bDMARDs) for at least one dosing cycle prior to surgery, when feasible, while maintaining non-biologic DMARDs in most cases. The decision must be individualized, taking into account the pharmacokinetics of each drug, the type of surgery, the patient’s comorbidities, and the activity of the underlying disease. Close coordination among rheumatologists, orthopedic surgeons, and infectious disease specialists is essential to minimize perioperative complications and optimize patient outcomes.

## 1. Introduction

The increased understanding of the systemic inflammatory processes underlying inflammatory rheumatic and musculoskeletal diseases (RMDs) has driven the development of disease-modifying antirheumatic drugs (DMARDs). These agents, by targeting crucial steps within the immune–inflammatory cascade, aim to improve disease control and prevent or at least slow the progression of joint and structural damage.

DMARDs are traditionally divided into two main categories in the management of musculoskeletal RMDs. The first group comprises conventional synthetic DMARDs (csDMARDs) such as methotrexate, leflunomide and sulfasalazine, which remain the cornerstone of first-line therapy for most inflammatory RMDs. When disease activity persists despite optimal csDMARD treatment, escalation to advanced DMARDs is indicated. This latter group includes biologic DMARDs (bDMARDs), targeting cytokines or immune cells such as Tumor Necrosis Factor-alpha (TNF-alpha), interleukins (IL-6, IL-1, IL-17, IL-23), or B and T lymphocytes, and targeted synthetic DMARDs (tsDMARDs), which act on intracellular signaling pathways such as Janus kinase (JAK) inhibition [1].

However, while these therapies have markedly improved long-term outcomes [2], their mechanisms of immune modulation inevitably interfere with physiological immune surveillance and host defense. This immunosuppression, although controlled, is associated with an increased susceptibility to infections, occasionally severe.

Therapies with DMARDs, particularly b- and ts-DMARDs, increase the risk of infection in patients with chronic arthritis [3]. The induced immunosuppression reduces host defenses against bacteria, viruses, and mycobacteria. Data from a systematic review by Singh et al., including more than 40,000 patients with RA from 106 RCTs, reported an annual absolute increase in the number of serious infections up to 55 cases per 1000 patients per year for therapies combined with biological treatments compared with patients treated with conventional synthetic DMARDs [4]. Respiratory infections are the most common, followed by gastrointestinal infections and sepsis. The risk is higher in elderly patients and those with comorbidities, requiring careful monitoring, vaccination prophylaxis, and temporary discontinuation of therapy in the presence of active infection [3].

The concern becomes particularly relevant in the perioperative setting, where infection risk is further heightened by surgical trauma and, in orthopedic surgery, by the possible presence of prosthetic or metallic implants [5].

Indeed, orthopedic infections are usually divided into two main groups: implant related and non-implant related infections, the former among the most feared especially if affecting joint replacements. The overall incidence of periprosthetic joint infection (PJI) for primary hip and knee replacement is between 0.3 and 1.9% but raises up to 10% for revision cases [6]. In the trauma setting, the infection rate is slightly higher (1 to 3%) following open reduction internal fixation occasionally reaching peaks of 50% in specific conditions like high-risk open fractures [7,8].

Among these, surgical site infections (SSIs) and PJIs represent some of the most severe postoperative complications, leading to increased morbidity, prolonged hospitalization, and, in some cases, the need for revision surgery. Since the early adoption of b- and tsDMARDs, initial reports recommended discontinuing these agents in the perioperative period [9]. Subsequent studies and clinical guidelines have largely confirmed this approach, emphasizing infection prevention as a key perioperative goal [10].

However, abrupt cessation of these therapies carries its own risks. Disease flares during the perioperative phase are common and may interfere with the postoperative recovery [11]. Therefore, balancing the need to minimize infection risk while preventing disease reactivation requires an individualized, evidence-based approach.

This review synthesizes current evidence and clinical recommendations on the perioperative management of biologic and immunomodulatory therapies in orthopedic surgery. It aims to provide a practical, decision-oriented framework by examining pharmacologic properties, infection risk profiles, and the benefits and drawbacks of continuing versus withholding these treatments around the time of surgery.

## 2. Methods

This narrative review was conducted to summarize and critically discuss the current evidence regarding the impact of biologic and targeted synthetic disease-modifying antirheumatic drugs (bDMARDs and tsDMARDs) on the risk, clinical presentation, and detection of infectious complications, with particular attention to postoperative infections and prosthetic joint infection.

A comprehensive literature search was performed using the electronic databases PubMed/MEDLINE, Google Scholar and Embase. The search covered publications from January 2000 to September 2025, reflecting the period during which biologic therapies became widely used in clinical practice. The last search was performed in September 2025.

The search strategy combined Medical Subject Headings (MeSH) terms and free-text keywords related to biologic therapies and infection. Key terms included, alone or in combination: “biologic DMARDs”, “targeted synthetic DMARDs”, “infection”, “prosthetic joint infection”, “postoperative infection”, “sepsis”, “C-reactive protein”, “erythrocyte sedimentation rate”, and “immunosuppression”. Boolean operators (AND/OR) were used to refine the search and increase sensitivity.

In addition, the reference lists of relevant articles were manually screened to identify further studies of interest. International guidelines and consensus statements from major scientific societies in rheumatology, orthopedics, and infectious diseases were also reviewed and included when pertinent to the perioperative management of patients receiving biologic therapies or to infection risk stratification.

Eligible sources included randomized controlled trials, observational studies (cohort and case–control studies), systematic reviews and meta-analyses, as well as high-quality narrative reviews addressing mechanisms of immunosuppression, infection risk, or diagnostic challenges associated with biologic therapies. Case reports and small case series were considered selectively when they provided clinically relevant insights, particularly regarding atypical presentations of infection or suppression of inflammatory markers. Non-English language articles and studies with insufficient methodological detail were excluded.

Given the narrative nature of this review, no formal assessment of study quality or risk of bias was performed. The available evidence was synthesized descriptively, with emphasis on clinically relevant findings, areas of consensus, and existing knowledge gaps.

## 3. Dimension of the Problem

The need for orthopedic interventions in patients on DMARDs is evolving: although advanced therapies have reduced the frequency of joint replacements, the aging population continues to contribute to a steady demand.

Patients with chronic inflammatory arthritis have a substantially higher likelihood of requiring joint replacement compared with the general population [12].

For example, rheumatoid arthritis (RA) alone affects approximately 0.24–1% of the global population, with females being usually two- to three-fold more affected than men [13]. Over 20% of these patients will require joint replacement surgery during their lifetime against the 8.1–11.6% lifetime risk of hip or knee replacement of the general population [14].

In a nationwide cohort, individuals with RA demonstrated a four-fold increased risk of undergoing total hip or knee arthroplasty, with the greatest excess risk observed at younger ages [15]. Similarly, population-based registry data show that patients with psoriatic arthritis experience nearly a two-fold higher incidence of major joint surgery over follow-up [16]. In ankylosing spondylitis, the risk of hip arthroplasty is particularly elevated, exceeding a ten-fold increase overall and markedly higher in young adults [17]. Collectively, these findings highlight the accelerated trajectory to end-stage joint damage in inflammatory arthritis relative to non-arthritic populations.

Even if, thanks to early diagnosis and DMARDs, the proportion of patients with RA undergoing total knee arthroplasty has dropped [18], their risk is still higher, especially in younger ages.

Additionally, many autoimmune diseases now have effective biologic treatments that allow patients to live longer and remain physically active [19], often increasing the likelihood of orthopedic injury or progressively long-term degenerative joint disease.

Biologic therapies have revolutionized the management of autoimmune and inflammatory disorders, offering targeted suppression of immune responses with fewer side effects than traditional immunosuppressants. However, these advantages are not costless, causing an impairment of host defenses, thus predisposing patients to infections, especially in the high-risk setting of orthopedic surgery.

Epidemiological studies have shown that patients receiving bDMARDs have a higher incidence of SSIs and PJIs compared to those on conventional DMARDs alone [20]. For example, in patients undergoing total joint arthroplasty, the infection rate in those continuing TNF inhibitors perioperatively is significantly elevated [21]. Furthermore, many biologics have long half-lives and mechanisms of action that persist well beyond their last dose, complicating efforts to mitigate risks by simply holding the drug before surgery.

SSIs and PJIs in patients with inflammatory rheumatic diseases are predominantly caused by skin commensals, reflecting perioperative contamination and biofilm-mediated persistence [22]. *Staphylococcus aureus* and coagulase-negative staphylococci (CoNS)—particularly *Staphylococcus epidermidis*—remain the most frequently isolated pathogens.

Less frequently, Gram-negative bacteria, including *Escherichia coli*, *Klebsiella* spp., *Enterobacter* spp., and *Pseudomonas aeruginosa*, account for a smaller but clinically relevant proportion of PJIs, especially in elderly, immunocompromised patients and following prolonged hospital stays or revision surgery. Anaerobes (e.g., *Cutibacterium acnes*) are increasingly recognized, particularly in shoulder arthroplasty, where they frequently cause indolent infections with minimal systemic signs.

In patients receiving biologic or targeted synthetic DMARDs (tsDMARDs), opportunistic and atypical pathogens must also be considered. These include mycobacteria (both *Mycobacterium tuberculosis* and non-tuberculous mycobacteria), fungal organisms (e.g., *Candida* spp.), and, rarely, Listeria monocytogenes or Nocardia. TNF inhibitors, IL-6 receptor inhibitors, and JAK inhibitors impair key immune pathways involved in intracellular pathogen control, thereby broadening the microbiological spectrum beyond typical pyogenic bacteria.

Biofilm formation is a central pathogenic mechanism in PJIs [23]. Following bacterial adherence to implant surfaces, microorganisms embed themselves within a self-produced extracellular polymeric matrix, which confers resistance to host immune responses and antimicrobial agents. Biofilm-associated bacteria exhibit reduced metabolic activity and altered gene expression, making them less susceptible to antibiotics and difficult to eradicate without implant removal.

Immunomodulatory therapies may indirectly favor biofilm persistence by impairing neutrophil recruitment, macrophage activation, and cytokine signaling, all of which are critical for early bacterial clearance. Once a mature biofilm is established, the infection often becomes chronic and refractory to conservative treatment, necessitating complex revision strategies [24].

PJIs are clinically classified based on timing and presentation. Acute infections (early postoperative or hematogenous) typically present with overt local signs such as erythema, swelling, warmth, wound drainage, and systemic symptoms including fever. These are most commonly caused by high-virulence organisms, such as *S. aureus* or Gram-negative bacilli [25].

In contrast, low-grade or chronic PJIs often present insidiously, with subtle pain, stiffness, or implant loosening and minimal inflammatory signs. These infections are frequently due to low-virulence, biofilm-forming organisms, such as CoNS or *Cutibacterium acnes* [26]. In patients treated with biologics—particularly IL-6 inhibitors—systemic inflammatory responses may be blunted, masking fever and suppressing CRP elevation, further delaying diagnosis [27].

Accurate microbiological diagnosis of SSIs and PJIs in immunomodulated patients is frequently difficult. Culture-negative infections occur in up to 42% of PJIs and are more common in patients who have received prior antibiotic therapy, even short courses [28]. Biologic-treated patients are particularly prone to atypical presentations and low bacterial burden, reducing culture sensitivity.

From an infectious disease perspective, patients with RMDs on biologic or tsDMARD therapy represent a high-risk population for both typical and atypical PJIs [29]. A high index of suspicion is required, especially for indolent infections caused by low-virulence organisms. Early multidisciplinary collaboration between orthopedic surgeons, rheumatologists, and infectious disease specialists is essential to guide diagnostic strategies, antimicrobial therapy, and decisions regarding implant retention or removal [30].

Ultimately, understanding the microbiological spectrum, biofilm biology, and immunologic impact of targeted therapies is critical to optimizing prevention, early detection, and management of SSIs and PJIs in this vulnerable patient population.

An appropriate prophylactic and therapeutic management of these kind of infections is widely debated, and evolving different pharmacological and not-pharmacological strategies proposed by scientific societies underline the uncertainties linked to the best therapeutic approach to effectively manage these difficult-to-treat infections.

From a societal perspective, the implications of postoperative infections in these patients are substantial, determining increased healthcare costs and longer hospital stays, but also undermining the functional goals of surgery [31]. This makes the question of how to manage these therapies before and after surgery a priority in clinical practice.

The present literature focused mainly on hip and knee surgeries with limited evidence for foot and ankle and upper limb. Elective spine surgery represents a further issue with a recent review which found a surgical site infection rate in the perioperative period of 26.0% and the need for a secondary revision surgery in up to 15.0% among patients who continued DMARD [32]. However, guidelines have been proposed with recommendation aligned to those by American College of Rheumatology (ACR) and the American Association of Hip and Knee Surgeons (AAHKS) [33,34,35,36,37]. For the purpose of this paper orthopedics procedures are not distinguished by anatomical district.

## 4. Commonly Used Drugs

**Figure 1:**
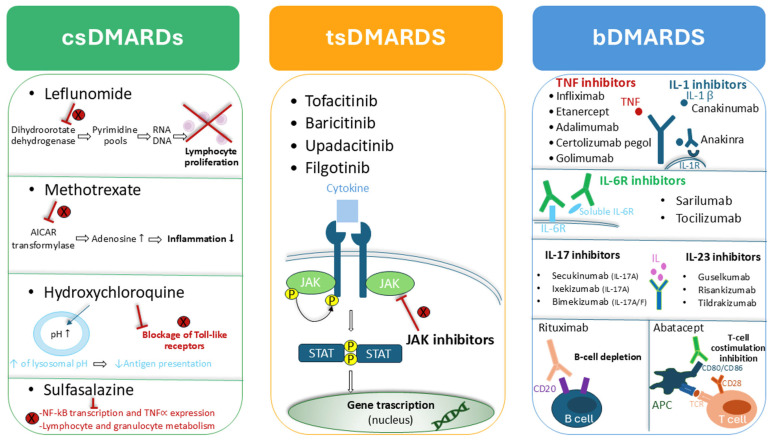
Classification and overview of the mechanisms of action of DMARDs used in rheumatic conditions.

Alongside non-steroidal anti-inflammatory drugs and glucocorticoids, commonly used DMARDs in rheumatic conditions include drugs with different mechanisms of action, as summarized in Figure 1.

### 4.1. Pharmacological Consideration

The pharmacokinetics of biologic agents determine how many and how long the drug remains in the body. Consequently, this information is essential to understand the optimal timing for discontinuation prior to surgery. Understanding the pharmacodynamics (PD) and pharmacokinetics (PK) of these agents is a key factor to guide perioperative planning. PK properties, including drug’s half-life and clearance, determine its circulating concentration and thus impact on the timing of therapy suspension and re-initiation in the perioperative period. Half-lives range from several days (etanercept: 3–5 days) to several weeks (rituximab: 5–78 days), with immunological effects sometimes persisting even longer.

From the PD point of view, these drugs are usually evaluated in terms of inducing a pharmacologic effect, taking in consideration their ability to reduce the inflammation burden and disease activity and progression. A wide range of biomarkers has been correlated in RA with the efficacy of DMARDs, and serum myeloid-related protein (MRP8/14), serum amyloid A (SAA), and C-reactive protein (CRP) have been proposed as pharmacodynamic markers [38]. However, the identification and validation of a specific PD marker is very complex to find in clinical setting and is still debated.

Thus, pharmacodynamic effects of DMARDs should also be considered, as their activity may persist even after the drug is no longer detectable in circulation. For example, Efe et al. reported a median duration of immunosuppression of 7 years after the last dose of rituximab, and 2% of the population study presented persistent B cell depletion [39], thereby exposing the patient to an infectious risk regardless of when the drug has been administered, especially in the case of repeated cycles. This is further complicated as the duration of B-cell depletion following rituximab treatment could be influenced by many factors, such as disease and patient characteristics and immune system dynamics [40]. In patients treated with rituximab, lymphocyte typing should therefore be considered.

Ideally, these medications should be held for one full dosing cycle before elective procedures, allowing enough time for the immunosuppressive effect to wane [41]. Of course, this exposes patients to the risk of flares which, in turn, may require corticosteroid therapy with increased risk for infections:

- Etanercept: Weekly dosing; often withheld for 1–2 week prior to surgery;
- Adalimumab: Biweekly dosing; withheld for 2–3 weeks;
- Infliximab: Administered every 6–8 weeks; surgery is timed at the end of the dosing cycle;
- Tocilizumab: Monthly IV or weekly SC, generally held, respectively, for 4 weeks or 1–2 weeks before surgery;
- Rituximab: Given every 6 months; timing surgery just before the next dose is ideal;
- Golimumab: Monthly dosing; generally withheld for 4 weeks before surgery;
- Secukinumab: Monthly dosing; withheld for one full dosing cycle (about 4 weeks);
- Ixekizumab: Monthly dosing; withheld for one full dosing cycle (about 4 weeks);
- Bimekizumab: Monthly dosing; withheld for one full dosing cycle (about 4 weeks);
- Guselkumab: Dosed every 8 weeks; surgery ideally timed at the end of the dosing cycle;
- Risankizumab: Dosed every 12 weeks; surgery ideally timed at the end of the dosing cycle;
- Anakinra: Administered daily; withheld for 2 days prior to surgery;
- Canakinumab: Monthly dosing; withheld for one full dosing cycle (about 4 weeks).

JAK inhibitors have shorter half-lives and can typically be held for 3–5 days preoperatively. However, they may also increase thromboembolic risk, adding another layer of complexity to perioperative planning [42].

When evaluating the safety profile of JAK inhibitors (JAKi) in patients with inflammatory arthritis, the concern extends beyond infectious complications, including prosthetic joint infections, to encompass thrombotic risk. Data from the ORAL Surveillance trial demonstrated an increased incidence of deep vein thrombosis (DVT) and pulmonary embolism in patients with RA treated with tofacitinib [43], prompting both the European Medicines Agency (EMA) and the Food and Drug Administration (FDA) to recommend restricting JAKi use in individuals at risk for DVT or major adverse cardiac events (MACE) to cases where biologic DMARDs have failed.

Venous thromboembolic events (VTE) risk, intrinsically increased in patients with RA, seems not to be further amplified after surgery, both in patients undergoing colectomy and orthopedic procedures, provided an adequate thromboembolic prophylaxis is administered [44,45].

In addition to these clinical observations, recent mechanistic evidence provides biological plausibility for such risk. Zavoriti and Miossec (2025) showed that while tofacitinib effectively reduced pro-inflammatory cytokine production—such as IFN-γ, IL-17A, and IL-6—in cocultures of peripheral blood mononuclear cells with synoviocytes or endothelial cells, it did not reverse the pro-thrombotic endothelial activation induced by inflammatory cytokines [46]. In particular, thrombomodulin expression remained suppressed, and tissue factor expression persisted, indicating that endothelial cells continued to exhibit a pro-coagulant phenotype despite JAK pathway inhibition [46].

These findings suggest that JAK inhibitors, while attenuating inflammation, may not fully normalize the vascular microenvironment and could contribute to a dose- and time-dependent increase in thrombotic risk, potentially linked to reduced IL-10 activity and impaired thrombomodulin regulation.

Table 1 summarizes the principal pharmacokinetic properties of DMARDs.

Table 2 shows a summary of perioperative management recommendations for commonly used drugs.

### 4.2. The Role of Therapeutic Drug Monitoring

Therapeutic Drug Monitoring (TDM) can be a valuable tool to confirm drug exposure. Yet, in this specific context, universally accepted reference concentrations to define effective therapeutic levels are still lacking for most of the agents considered in each specific clinical setting, although some evidence has begun to emerge for a few of them. TDM of TNF inhibitors has emerged as a promising strategy to optimize treatment in RA. Evidence from clinical studies supports its role in individualizing therapy and improving outcomes. Mulleman et al. (2009) demonstrated that monitoring infliximab concentrations facilitates better disease control by enabling dose adjustments tailored to serum levels and clinical response [66]. More recent real-world data on adalimumab confirmed a clear relationship between trough levels, antidrug antibodies, and disease activity, underscoring the potential of TDM to identify suboptimal exposure and immunogenicity as key drivers of therapeutic failure [67]. Furthermore, the work by Pouw et al. (2015) delineated the adalimumab-concentration–effect curve, establishing exposure thresholds associated with clinical efficacy [68]. While several studies have shown correlations between drug concentrations, antidrug antibodies, and clinical response, these results have not yet translated into widespread clinical implementation and recommendations for treatment or for the perioperative management. Similarly, further prospective studies are needed to clarify whether a correlation exists between bDMARDs serum concentrations and an increased incidence of infection. Furthermore, this evaluation is complicated as disease activity and severity themselves represent risk factors for infection [11]. Moreover, TDM could be a useful tool to employ in clinical practice to evaluate when to stop DMARD treatment by considering drug concentrations, rather than a fixed time interval suspension. This could theoretically be a valuable strategy to balance both risk of infection and disease flares in the perioperative setting, particularly for drugs with high PK inter-individual variability. Although to our knowledge this approach has not been investigated in orthopedic surgery, a review in inflammatory bowel disease evidenced the potential role of TDM in the surgical patient [69].

While the literature on TDM in this clinical setting mainly focuses on TNF inhibitors, the extrapolation to other biopharmaceuticals remains still unclear [70].

On top of evaluation of DMARDs concentrations, a more effective activity of TDM focused on this clinical setting should always take in account renal and hepatic function, concurrent medications (e.g., corticosteroids), and the nature of the planned surgery. Furthermore, high-risk surgeries, such as those involving prosthetic implants or spinal instrumentation, warrant stricter immunosuppression management.

Overall, these data suggest that TDM cannot currently be routinely recommended to guide the perioperative management of DMARDs due to a lack of validated thresholds for infection risk. Further clinical trials are required to generate evidence filling this gap of knowledges, especially in case of high risk-scenarios when therapeutic target concentrations of DMARDs represent a game-changer in the clinical management of patients. Secondarily, due the severity of these diseases and the high costs related to DMARDs therapies, at least until biosimilars enter the market, TDM of this kind of drugs could be employed as a tool for optimizing long-term disease control and for testing patients’ therapeutic compliance in case of clinical failure.

## 5. Risk Factors Enhancing Infection Risk

Patients with RMDs undergoing orthopedic procedures have both disease- and treatment-related risks for infection, in addition to those intrinsic to surgery.

Chronic inflammation and immune dysregulation, typical of the underlying diseases, like RA or psoriatic arthritis, together with comorbidities, including diabetes, malnutrition, obesity, chronic kidney disease and smoking, increase infection susceptibility [71]. The frailty of these patients, worsened by the hypomobility due to end-stage arthritis, further diminishes the immune reserve. The awareness for infection risk, typical of those conditions, cannot prevent from proceeding with the surgeries but requires extreme caution in the perioperative phase.

Available data indicate that prosthetic joint infection risk in patients with inflammatory arthritis varies according to age, disease duration, comorbidities, active disease, corticosteroid exposure, and prior infection history. In particular, patients older than 65 years, with long disease duration, active joint disease, prolonged corticosteroid use, and a history of surgery-associated infections should be considered at higher risk.

On the other side, treatment-related risk factors are strictly correlated to the action mechanism of each DMARDs, interfering with macrophage and neutrophil activation, impairing granuloma formation or depleting antibody-mediated immunity.

Finally surgical and perioperative factors should be considered. In particular, longer surgeries, requiring the use of implants, like joint replacements and spinal fusion, are those at higher risk. The timing of the procedure is crucial in the management of risk factors. Traumatic and elective conditions deeply differ in the approach. If joint replacement usually allows a delay to optimize the patient, that is not the case for traumatic surgeries, in which the window of opportunity to proceed is much narrower. More than 25 modifiable risk factors have been correlated to an increased risk for SSI and PJI, and most of them are chronic conditions, manageable but not solvable in few days [72]. Certainly, strict adherence to preoperative antibiotic prophylaxis protocols is recommended in terms of timing, molecular choice and doses, and it is fully independent from chronic medical conditions or acute surgical settings. Main risk factors and their impact on infection risk are summarized in Table 3.

Therefore, the perioperative management of biologic DMARDs, including treatment interruption, should be individualized, balancing infection risk against the risk of disease flare.

## 6. Impact of Biologic Therapies on the Course and Detection of Infectious Complications

Biologic and targeted synthetic disease-modifying antirheumatic drugs (bDMARDs and tsDMARDs) not only increase susceptibility to infections but may also substantially alter their clinical course and detectability. By selectively suppressing key immune mechanisms—including cytokine signaling and the activity of neutrophils and macrophages—these agents can blunt the inflammatory response that typically accompanies infection. As a result, classic clinical features such as fever, erythema, swelling, and pain may be attenuated or absent, leading to atypical or delayed presentations. This is particularly relevant in the postoperative setting, where early manifestations of prosthetic joint infection or systemic infections such as sepsis may be masked, resulting in delayed diagnosis and poorer clinical outcomes.

A major consequence of immune modulation by biologic therapies is the reduced diagnostic utility of conventional inflammatory markers. Acute-phase reactants such as C-reactive protein (CRP), erythrocyte sedimentation rate (ESR), and fibrinogen are routinely used to support the diagnosis of infection and to monitor response to treatment; however, several biologic agents directly interfere with the cytokine pathways responsible for their production. This effect is most pronounced with interleukin-6 (IL-6) inhibitors, such as tocilizumab. IL-6 plays a pivotal role in hepatic synthesis of acute-phase proteins, and its blockade can result in profoundly suppressed CRP and ESR levels even in the presence of severe bacterial infections or sepsis. Numerous reports have documented microbiologically confirmed infections occurring in patients treated with tocilizumab despite normal or minimally elevated inflammatory indices, highlighting a significant dissociation between laboratory findings and clinical reality [73].

The suppression of inflammatory markers not only compromises early diagnosis but also limits their usefulness for monitoring therapeutic response. Persistently low or declining CRP values in patients receiving IL-6 inhibitors may reflect pharmacologic effects rather than effective infection control, potentially leading to delayed recognition of treatment failure or inappropriate modification of antimicrobial therapy [27]. Although less pronounced, similar alterations in inflammatory and febrile responses have also been described with other biologic agents, including TNF-α inhibitors and Janus kinase (JAK) inhibitors. Consequently, an inflammatory profile within the normal range does not reliably exclude the presence of an active infection in patients receiving biologic therapy.

For these reasons, a high index of clinical suspicion and heightened vigilance are mandatory in patients considered at increased risk. Clinical assessment should take precedence over laboratory parameters, and early use of microbiological investigations and imaging studies should be strongly considered when infection is suspected. In high-risk contexts, such as the perioperative period or in patients presenting with unexplained pain, functional deterioration, or subtle systemic symptoms, timely invasive diagnostic procedures should not be deferred solely on the basis of reassuring inflammatory markers.

## 7. Risks of Continuing

The current state of the art is that continuing biologic or immunomodulatory therapy in the perioperative period may lead to surgical site infections (SSIs), including prosthetic joint infections (PJIs), delayed wound healing and systemic infections, from pneumonia to sepsis. One of the main mechanisms involved is the impairment of neutrophil cells function, thus reducing early defense mechanisms. Also, the alteration of the cytokines patterns can affect the normal immunological cascade involved in the normal healing process. Several retrospective studies and meta-analyses have documented these risks, though heterogeneity in study design and patient population complicates definitive conclusions. Nevertheless, the trend suggests that ongoing biologic therapy is associated with higher infection-related complications.

Although incidence of PJI in orthopedics is pretty low, occurring in 1% to 2% of primary and in 4% of revision arthroplasties, the risk is intrinsic to the surgical procedure, and, despite all the efforts in prevention, it is always present [74]. PJIs represent a devastating complication of joint replacement, with consequences for the patients, caregivers and society, including a not negligible higher risk for mortality. The risk factors include perioperative conditions, both modifiable and non-modifiable, related to the host and to surgery. Among these factors are also adequate infection prevention measures, which require the appropriate and judicious use of antimicrobial therapy, with the dual aim of preventing the potential onset of bacterial infection and avoiding the promotion of antimicrobial resistance. Among the most widely proposed strategies to prevent infections at the perisurgical site are screening for *Staphylococcus aureus* carriage with subsequent decolonization, preoperative skin cleansing with chlorhexidine or washing with soap at least the night before surgery, and the local delivery of antimicrobial agents into the wound site [75]. The antimicrobial agents most commonly used for prophylactic purposes primarily include cefazolin, to be administered within approximately 60 min before surgery; in cases of MRSA colonization, the addition of vancomycin is also recommended [75,76,77]. However, uncertainty remains regarding the optimal duration of antibiotic prophylaxis, particularly in populations at high risk of PJIs, and a phase IV clinical trial is currently evaluating this issue, with results expected in June 2026 (NCT04297592). When antimicrobial therapy is required for therapeutic purposes, treatment should be pathogen-directed whenever the causative organism is known, whereas in empirical settings the choice of agents should be guided by the type and site of infection (e.g., prosthesis retention), as well as by the suspected pathogen. The main antimicrobials indicated include rifampicin, penicillins, cephalosporins, carbapenems, oxazolidinones, and fluoroquinolones, or vancomycin or daptomycin in cases of MRSA or MRSE infection [78,79]. New interesting pharmacological approaches derive also from dalbavancin and oritavancin in case of chronic infections, although their use is still off-label in this setting [80,81]. Given the site of infection and the severity of the infectious course, often associated with multiple potential complications, a targeted and individualized antimicrobial treatment is increasingly recommended, tailored to both the pathogen and patient-specific characteristics. This personalized approach may also be supported by TDM of the administered antimicrobials, considering the sometimes unpredictable nature of their PK/PD profiles [80,81,82,83,84,85].

Genetic predisposition and a personal or a familiar history of PJI are non-modifiable and need to be considered. Among patients’ risk factors, smoking, alcohol and intravenous drug use, poor oral hygiene, diabetes and nutritional issues are considered as modifiable or at least partially amendable [86]. As stated before, further issue is the intake of immunomodulant drugs, like glucocorticoids and DMARDs [87]. Prednisone prescription is reported to have an OR of 1.59 in predicting post operative PJI [88] while bDMARDS raise the OR to 5.69 for SSI, and further over 9.0 if TNF-alfa blockers (infliximab and etanercept) are involved [89], with knees being reported at higher risk [90].

The higher incidence for SSI (OR 1.39, 95% CI 1.12–1.72), without a statistically significant difference in delayed wound healing, has been highlighted for patients who continued bDMARDS, in a recent meta-analysis by Imam, including a total population of 19,021 individuals undergoing various orthopedic surgeries [5]. These data are in contrast with a previous meta-analysis by van Duren, which reported a non-significant increase in SSI and wound complications with a significantly lower risk for disease flares in those patients continuing bDMADS [91].

## 8. Risks of Stopping

The reported higher risk for SSI has determined the habits to stop bDMARDS, but discontinuation is not harmless. In the setting of a stable disease, the sudden interruption of therapy may lead to disease flares, which are acute worsening of inflammation and pain associated to the disease. These flares are common after joint replacement and reported in up to 63% within 6 weeks of surgery, with a third rated as severe [92]. There is some evidence suggesting that those flares could be independent from the drugs’ management but due to the perioperative stress. Of course, these symptoms may interfere with rehabilitation and lengthen the hospital stay.

Together with that delay in autonomy recovery, also, the risk for hospital readmission may raise.

There is also a not negligible risk that immunologic effects of many biologics persist well beyond the last administered dose, meaning immune modulation may continue despite temporary cessation. Moreover, surgical stress itself induces immune dysregulation, which may outweigh any short-term benefit gained from stopping therapy.

Discontinuation of biologics can also precipitate rheumatoid disease flares, requiring corticosteroids, which are a well-established and dose-dependent risk factor for postoperative infection.

Consequently, the net effect of stopping biologics may be neutral or even unfavorable with respect to infection risk, while increasing the likelihood of inflammatory complications.

Furthermore, the clinical presentation with pain and swollen joints and a suggestive haemato-chemical pattern (raised CRP and ESR) may mimic a SSI, raising concerns on the effective nature of the flare [93].

The differential diagnosis between a rheumatic flare and postoperative infection is particularly difficult because of the substantial clinical overlap between the two conditions. Low-grade fever and malaise may be also present in either, and normal postoperative inflammation further obscures interpretation.

Laboratory markers lack specificity with CRP and ESR usually risen after surgery as part of the normal inflammatory response but also elevated during flares and infection. Furthermore, baseline inflammatory markers may already be high in rheumatic patients, making postoperative trends difficult to interpret. Leukocyte counts are similarly unreliable due to surgical stress, steroid therapy, or immunosuppression.

Imaging findings are non-specific as well, as postoperative edema, synovitis, and effusions are expected and overlap with findings seen in both flares and infection, limiting the diagnostic value of ultrasound or MRI in the early postoperative period.

Finally, synovial fluid analysis may still be ambiguous. Inflammatory synovial fluid with elevated white cell counts and neutrophil predominance can be seen in both conditions, and low-grade or partially treated infections may yield borderline counts or negative cultures.

Risks of misclassification are relevant in terms of treating a flare as an infection leading to unnecessary antibiotics, delay in restarting immunosuppressive therapy and prolonged pain and disability. On the other hand, undertreating an infection may determine sepsis, prosthetic failure and chronic infection.

Together, these factors make early distinction challenging and force clinicians to prioritize exclusion of infection before diagnosing a rheumatic flare, often requiring repeated assessments, invasive testing, and multidisciplinary decision-making.

## 9. Literature and Perspectives

Several guidelines, including those from the ACR and the British Society for Rheumatology (BSR), offer consensus-based recommendations. These generally support holding biologics for one dosing cycle before elective surgery and resuming postoperatively once the wound shows signs of healing and no infection is present. More recent literature supports individualized decision-making based on disease activity, drug half-life, and surgical risk level.

The current literature seems to agree in stopping DMARDs in the perioperative period to reduce the risk for SSIs. In orthopedics, SSIs go from wound healing delay to PJIs, which represent a disabling and expansive complication with huge individual and social consequences. Great efforts are daily performed in the optimization of the patient waiting for a scheduled operation to prevent PJI. Anemia, hyperglycemia, overweight or malnutrition, and smoking status are all factors evaluated and possibly corrected. DMARDs for rheumatoid patients represent a risk factor, as well. On the other side, a too early suspension or a delayed restart raises the risk for flare-up of the disease, with pain, stiffness and functional limitation interfering with the postoperative recovery. These flares-up may be severe, thus compromising both years of disease stability and postoperative rehabilitation. This subtle balance raises the question if DMARDs suspension is mandatory. At the recent 3rd Meeting of the International Consensus Meeting held in Istanbul, the recommendation with strong literature evidence has been “…biological and targeted synthetic DMARDs should be stopped prior to major orthopedic procedures. The exact time to withhold the drugs depends on the half-life of these drugs” [94]. From 2022 to 2025, the paradigm seems to have changed. If ACR/AAHKS recommendations focused on the drugs with medications to continue and to withhold, recently the underlying rheumatic condition seems to play a central role, involving the treating rheumatologist in a shared approach.

Nowadays a multi-center, pragmatic randomized clinical trial, called PERISCOPE, is investigating the outcomes of elective orthopedics procedures (soft tissue surgery, joint replacement or another metal-work implantation) performed without the perioperative holiday period. Clinical self-reported outcomes and local or systemic complications are assessed in participants either stopping or continuing bDMARDS [95].

Moreover, this constantly happens when approaching traumatic fracture in an urgent/acute setting. In these cases, the timeframe between trauma and surgical treatment is not adequate to allow the usual suspension, if not at the cost of further delay in the fracture fixation. In those non-elective patients, there is a lack of evidence for the optimal management of bDMARDS, at least for the postoperative resumption as usually the preoperative approach is bound to the need of fixation.

Future directions include the development of predictive models to stratify infection risk, incorporating biomarkers and patient-specific data. Ongoing trials are exploring the feasibility of shorter discontinuation windows and postoperative bridging strategies with non-biologic DMARDs.

After an initial response to monotherapy for RA, a proportion of patients could fail or develop resistance, thus requiring the introduction of a combined approach with additional drugs. The cumulative risk of multiple contemporary agents needs to be investigated to understand if there is a proportional or exponential increase in the risk for infections, as multiple immunological pathways could be involved.

Several questions remain unanswered. For example, do the DMARDs raise the risk of complications including infection, or is it the rheumatological disease itself? Furthermore, is the need for metalwork implantation a crucial aspect to consider in the decision-making process? Could the Therapeutic Drug Monitoring of bDMARDs be used for individualized perioperative patient management?

A key aspect is also to define who is in charge for the final decision. A national survey in UK has been conducted, and interestingly a different point of view emerged. Most of rheumatologists felt more of the responsibility of the management with a very limited role for the surgeon. On the others’ side, more than half of the surgeons involved and a third of the rheumatologists acknowledged the need for a joint decision [96].

Multidisciplinary collaboration remains crucial. Preoperative planning should involve rheumatologists, orthopedic surgeons, anesthesiologists, and infectious disease specialists. Enhanced recovery protocols, including optimized nutrition and glucose control, may further mitigate infection risks.

Ultimately, balancing the risks of infection against the risks of disease flare is not a binary decision but a spectrum, requiring careful deliberation. As evidence grows, personalized medicine will likely take a central role in perioperative management for this complex patient population.

It is not just a matter of “to stop or not to stop” a drug. This is a piece of a bigger picture in which a patient, suffering from a chronic condition and undergoing surgical procedure, is the key factor to be considered.

## 10. Conclusions

The perioperative management of biologic and immunomodulatory therapies in orthopedic surgery represents a complex clinical balance between infection prevention and disease control. Biologic and targeted synthetic DMARDs have transformed outcomes in inflammatory arthritis but confer an increased susceptibility to surgical site and prosthetic joint infections, particularly in high-risk procedures involving prosthetic implants.

Current evidence supports temporary discontinuation of DMARDs prior to major orthopedic surgery, generally for one dosing cycle, while maintaining conventional synthetic DMARDs in selected patients. However, drug pharmacokinetics, persistent immunologic effects, and patient-specific factors limit the protective impact of uniform withdrawal strategies. Conversely, treatment interruption carries a substantial risk of perioperative disease flares, functional impairment, delayed rehabilitation, and increased corticosteroid exposure, which itself heightens infection risk. The diagnostic overlap between inflammatory flares and postoperative infection further complicates postoperative management.

Emerging data suggest that rigid protocols may be less effective than individualized, risk-adapted strategies. Multidisciplinary collaboration between rheumatologists, orthopedic surgeons, and infectious disease specialists is therefore essential. Ongoing trials and future research into biomarkers, Therapeutic Drug Monitoring, and predictive risk models may refine decision-making.

Ultimately, perioperative DMARD management should move beyond a binary “stop versus continue” approach toward personalized care centered on the patient, the disease and the surgical context.

## Figures and Tables

**Table 1:** Pharmacokinetic properties of DMARDs relevant to therapy suspension during perioperative period.

Drug	Route of Administration	Half-Life t_1/2_	Metabolism	Elimination
Methotrexate [47]	Oral/SC ^1^/IM ^2^	3–17 h	Hepatic	Renal/feces
Sulfasalazine [48]	Oral	~7.6 h	Intestinal flora/Hepatic	Renal/feces
Leflunomide [49]	Oral	~2 weeks	Hepatic	Feces/renal
Hydroxychloroquine [50]	Oral	Terminal ~40–50 days	Hepatic	Renal
Tofacitinib [51]	Oral	~3 h *	Hepatic	Renal
Baricitinib [52]	Oral	~12.5 h	Hepatic	Renal/feces
Upadacitinib [53]	Oral	Terminal 9–14 h	Hepatic	Feces/renal
Anakinra [54]	SC	Terminal 4–6 h	-	Renal
Canakinumab [55]	SC	Terminal 26 days	Proteolytic degradation	
Infliximab [56]	IV ^3^/SC	~8–9.5 days		
Etanercept [57]	SC	~70 h		
Adalimumab [58]	SC	Terminal ~2 weeks		
Certolizumab pegol [59]	SC	Terminal ~14 days		
Golimumab [60]	IV/SC	Terminal ~12 days		
Secukinumab [61]	IV/SC	18–46 days IV		
Ixekizumab [62]	SC	13 days		
Tocilizumab [63]	IV/SC	Up to 16 days, concentration dependent		
Rituximab [64]	IV	Terminal 8.58–35.9 days		
Abatacept [65]	IV/SC	8–25 days IV		

^1^ SC = Subcutaneous; ^2^ IM = Intramuscular; ^3^ IV = Intravenous; * Depending on release formulation.

**Table 2:** Summary of perioperative management recommendations by drug.

Drug	Dosing Schedule	Preoperative Withholding	Postoperative Restart	Notes
Etanercept	Weekly SC	Hold 1–2 weeks prior to surgery	Resume once wound shows adequate healing and no infection	Short half-life; low immunosuppressive carryover
Adalimumab	Every 2 weeks SC	Hold 2–3 weeks prior to surgery	Resume once wound shows adequate healing and no infection	Timing based on dosing interval
Infliximab	Every 6–8 weeks IV	Schedule surgery at end of dosing cycle	Resume at next planned dose if no infection	High immunosuppressive effect; avoid early restart
Tocilizumab	Monthly IV/weekly SC	Hold 4 weeks (IV) or 1–2 weeks (SC)	Resume once wound shows adequate healing and no infection	May blunt CRP response; monitor carefully
Rituximab	Every 6 months IV	Schedule surgery just before next dose	Resume at next cycle if no infection	Immunosuppressive effect may persist for months; monitor B-cell counts
Golimumab	Monthly SC/IV	Hold 4 weeks prior to surgery	Resume once wound shows adequate healing and no infection	Standard biologic precautions
Secukinumab	Monthly SC/IV	Hold 1 full dosing cycle (~4 weeks)	Resume once wound shows adequate healing and no infection	IL-17 inhibitor; limited data for urgent surgery
Ixekizumab	Monthly SC	Hold 1 full dosing cycle (~4 weeks)	Resume once wound shows adequate healing and no infection	Similar to secukinumab
Bimekizumab	Monthly SC	Hold 1 full dosing cycle (~4 weeks)	Resume once wound shows adequate healing and no infection	IL-17A/F inhibition; follow same precautions
Guselkumab	Every 8 weeks SC	Schedule surgery at end of dosing cycle	Resume at next planned dose	IL-23 inhibitor; limited perioperative data
Risankizumab	Every 12 weeks SC	Schedule surgery at end of dosing cycle	Resume at next planned dose	Long dosing interval; consider delayed restart
Anakinra	Daily SC	Schedule surgery after 2 days	Resume once wound shows adequate healing and no infection	IL-1 receptor antagonist; short half-life (4 to 6 h)
Canakinumab	Monthly SC	Hold 1 full dosing cycle (~4 weeks)	Resume once wound shows adequate healing and no infection	Fully human IgG1k monoclonal antibody for IL-1β

**Table 3:** Risk factors and their impact on infection risk by category.

Category	Risk Factor	Impact on Infection Risk	Implications for Continuing vs. Withholding DMARDs
Patient-related	Age > 65 years	Increased risk of SSI and PJI	Favors withholding b/tsDMARDs
	Diabetes mellitus	Higher risk of infection and impaired wound healing	Supports withholding; optimize glycemic control
	Obesity or malnutrition	Increased SSI and delayed healing	Strengthens indication to withhold
	Smoking	Higher SSI and wound complications	Withholding recommended; smoking cessation
	Chronic kidney disease/multiple comorbidities	Increased susceptibility to infection	Conservative approach → withhold
	History of prior surgical infection	High risk of recurrence	Strong indication to withhold
Disease-related	High disease activity	Increased infection risk and flare probability	Complex decision; balance flare vs. infection risk
	Long disease duration	Greater immune frailty	Favors withholding
	Systemic inflammatory arthritis (RA, PsA)	Higher PJI risk vs. general population	Individualized approach required
	Chronic or high-dose corticosteroid use	Dose-dependent infection risk	Minimize steroids; withhold DMARDs if feasible
Treatment-related	Biologic DMARDs	Increased SSI and PJI risk	Generally withhold for one dosing cycle
	Rituximab	Prolonged immunosuppression	Surgical timing more relevant than brief drug holiday
	JAK inhibitors	Infection risk ± thromboembolic risk	Withhold 3–5 days preoperatively
	Combination immunosuppressive therapy	Cumulative infection risk	Favors withholding
	Conventional synthetic DMARDs	Modest infection risk	Often safe to continue
Surgery-related	Major orthopedic surgery	Increased SSI risk	Favors withholding
	Prosthetic implantation/metalwork	High PJI risk	Strong indication to withhold
	Prolonged or complex procedures	Increased bacterial exposure	Withholding recommended
	Urgent or trauma surgery	No time for preop suspension	Reassess postoperative restart
	Early, uncomplicated wound healing	Lower infection risk	Allows earlier DMARD resumption

## Data Availability

No new data were created or analyzed in this study. Data sharing is not applicable to this article.

## Abbreviations

The following abbreviations are used in this manuscript:

SSI	Surgical Site Infection
DMARDS	Disease-Modifying Antirheumatic Drugs
RMDs	Rheumatic and Musculoskeletal Diseases
TNF	Tumor Necrosis Factor
JAKi	Janus Kinase Inhibitors
PJI	Periprosthetic Joint Infection
RA	Rheumatoid Arthritis
ACR	American College of Rheumatology
AAHKS	American Association of Hip and Knee Surgeons
FDA	Food and Drug Administration
DVT	Deep Vein Thrombosis
EMA	European Medicines Agency
MACE	Major Adverse Cardiac Events
VTE	Venous Thromboembolic Events
TDM	Therapeutic Drug Monitoring
BSR	British Society of Rheumatology
